# Ethnoveterinary Therapeutic Practices and Conservation Status of the Medicinal Flora of Chamla Valley, Khyber Pakhtunkhwa, Pakistan

**DOI:** 10.3389/fvets.2019.00122

**Published:** 2019-05-16

**Authors:** Khalid Khan, Inayat Ur Rahman, Eduardo Soares Calixto, Niaz Ali, Farhana Ijaz

**Affiliations:** ^1^Department of Botany, Hazara University, Mansehra, Pakistan; ^2^William L. Brown Center, Missouri Botanical Garden, St. Louis, MO, United States; ^3^Department of Biology, University of São Paulo, São Paulo, Brazil; ^4^Department of Biology, University of Missouri, St. Louis, MO, United States

**Keywords:** traditional knowledge, medicinal plants, animal care, ethnomedicinal quantification, statistical indices, conservation, Buner, Pakistan

## Abstract

Domestic animals play a very important role in the human civilization. Besides human being, plants are used as medicines for many domestic animals. The therapeutic practices are very common among the tribes of Chamla, rich in ethnoveterinary medicinal plants. Due to poor availability of modern healthcare facilities and poverty of indigenous people, they depend on local medicinal plants for the healthcare of their domestic animals. This study is the first attempt to document the indigenous knowledge and evaluate the conservation status of medicinal plants and practices of herbal remedies by the local people of Chamla Valley in the treatment of their livestock. Semi-structured questionnaire was used and 120 local inhabitants were interviewed to note the traditional practices regarding plant species uses. Well-known statistical indices, Use Value formula and Relative Frequency Citations were used for quantification of the recorded data. It was observed that 50 medicinal plants belonging to 38 families were reported, where Poaceae was the most cited. The common livestock are goats, sheep, buffalos, cows, bulls, and donkeys. Most of the herbs, which are used in livestock treatment, are wild and few plants are cultivated. The common livestock diseases are red water, 3 days sickness, diarrhea, tympany, and indigestion among others. Most of the plants are used in fresh condition. According to the results, *Brassica nigra* was used for placenta retention, *Butea monosperma* for constipation, *Calotropis procera* for indigestion and 3 days sickness. *Canabis sativa, Cedrella serrata, Allium sativum*, and *Origanum vulgare* were used for fever. The traditional plant collection techniques have resulted in huge losses of these valuable plant resources. The ethnobotanical conservation assessment revealed that due to increased exploitation and un-sustainable harvesting, 49% of these economically valued medicinal plant species are decreasing in last 30 years. Some of the plants are only present on high altitudes while they had been finished in the foothills like *Paeonia emodi and Berberis lycium*. Lack of scientific knowledge, ignorance, poverty, and joblessness, as well as land development, construction and fires, add more pressure on flora and fauna of the area and various species are under the threat of extinction.

## Introduction

Livestock keeping is one of the vital economic sources forming integral part of the traditional tribal community. Animals are a source of calories in the form of meat, milk, and its derivatives for the livelihood of local people and also they are a source of earning. Livestock plays an important role in national economy of Pakistan (1), since the majority of the people of the current study area depends on agriculture and livestock production. Livestock is the largest contributor to overall agriculture value. The livestock owners have to rely on the herbal medicinal plant recipes being inherited by their predecessors generation to generation.

Dependency and sustainability of man and animal lives has been revolving around plants through their uses as foods, fibers, and shelter, as well as to control and ease diseases, which is an ancient and reliable practice (2). Ailments and medicinal plants vary in the world, hence their nature, frequency, and methods of administration can change in relation to geography, time and knowledge. Indigenously, different plants have been used to cure a disease or several diseases at a time, but toward the middle of 20th century the contribution of medicinal plants to medicine was reduced by approximately one fourth as research and development favored the use of synthetic chemicals. Now this trend is reversing once again in favor of plants, as they have been discovered to possess natural products that are chemically balanced, effective, less injurious with none or much less side effects (3). Pakistan, China, and India are the supreme users of medicinal plants. Their traditional practices of plant remedies date back at least 7,000 years (1). The influences of traditional medicine upon western medicine have been both indirect and direct (4).

Ethnoveterinary medicine, the scientific term for traditional animal health care, provides low-cost alternatives to allopathic drugs (2, 5). Research in such field is often undertaken as part of a community-based approach that serves to improve animal health and provide basic veterinary services in rural areas (6, 7). In addition, ethnoveterinary medicines cover people's knowledge, skills, methods, practices, and beliefs about the care of their animals (2). In many poor rural areas ethnoveterinary medicine can play an important role in animal production and livelihood development and often becomes the only available means for farmers to treat ill animals (8). These medicines provide valuable alternatives to and complement western-style veterinary medicine. During the late 1930, it was observed that cattle fed on spoiled sweet clover died from hemorrhage. On examination it was finally established that this hemorrhagic effect was due to a chemical, dicoumarin now known under the trademark dicoumarol. In 1941, synthesis of this anticoagulant agent was done by Link Stabmann and Huebner and then, it was beneficently employed (4).

Therefore, it is extremely necessary to document and disseminate indigenous knowledge in order to help and share the different uses of plants as animal health care and to promote different conservation measures. Thus, the aim of this study was to evaluate the Ethnoveterinary therapeutic practices and conservation status of the medicinal flora of Chamla Valley, Khyber Pakhtunkhwa, Pakistan. This is the first attempt to document the indigenous knowledge and evaluate the conservation status of medicinal plants and practices of herbal remedies by the local people of Chamla Valley in the treatment of their livestock.

## Materials and Methods

### Study Area

Buner District (34°26′34.83″N, 72°29′57.58″E) is located in Khyber Pakhtunkhwa Province, Pakistan and it was a part of Swat up to 1969. The region presents an area of 1,865 km^2^. Chamla valley has an area of 49 km^2^ and elevation of 670 m. The valley is drained by Chamla River which finally joins Indus River (9). Buner District is administratively divided into six tehsils namely: Daggar, Gadezi, Chagharzai, Gagra, Chamla, and Totalai. Elevation varies from 366 meters in Totalai in the south to 2,911 meters of the Dosara peak in the North. Total population of Buner district is more than 0.9 million.

This region was chosen, because economically the majority of people are poor and agriculture and livestock is the main source of livelihood of the population, where they have their own treatment system for most of the diseases of their domestic animals. Therefore, this region is an excellent study model for the documentation and dissemination of indigenous knowledge in order to help and share the different uses of plants in animal health care. The main livestock include cattle, buffalos, sheep, goats, camels, horses, asses, and mules.

### Data Collection

Twelve villages were randomly selected from the upper, middle, and lower areas. In each village, randomly 10 inhabitants were interviewed. Out of these ten, five were common persons involved in farming practices while five were the expert in the use of these plants for the treatment of livestock diseases.

The work was initiated in the result of discussion with experts of knowledge of medicinal plants and experts of study area for identifying and conserving this precious source of natural resources. In detail, the study was conducted in the following steps: *(i) Collection of baseline information and Sampling design*—Field surveys were carried out and interviews of the local informants were conducted. During field trips, people including local traditional practitioners, veterinary doctors, farmers, and other local respondents were interviewed on random basis for the traditional uses of indigenous plants in curing ethnoveterinary diseases. Semi-structured questionnaire was used to note the traditional knowledge regarding plant species uses [sensu (10)]; *(ii) Identification*—The samples of the plants were identified in different departments, such as PCSIR (Peshawar), Pakistan Forest Institute (Peshawar), and Department of Botany, University of Peshawar. After filed surveys, different analyzes related to the use of the plants in the animal health care were used (see Statistical analysis below) to observe the relevance of each species in the animal health care.

### Statistical Analyses

*Use Value (UV*_*i*_*)*. UV_i_, the Use Value of a plant species was calculated by using the formula [8];

$$\begin{array}{l} \text{U}{\text{V}}_{\text{i}}=\Sigma {\text{U}}_{\text{i}}/{\text{N}}_{\text{i}} \end{array}$$

U_i_ = Use reports cited for a particular plant species by each respondent and N_i_ = Total informants interviewed for a particular plant species.

*Relative frequency of citations (RFCs)*. RFCs index was used to assess the traditional uses and medicinal value of each species in the area (11).

$$\begin{array}{l} \text{RFCs}=\text{FCs}/\text{N} \end{array}$$

FCs = No. of local respondents who use the taxa traditionally and *N* is the total number of respondents of in the study (in this study, *n* = 120).

Multivariate ordination analyses, “principal components analysis” (PCA) and species response curve (SRC), were used to evaluate differences in the conservation status and nature of the plant species reported. A species response curve (SRC) was drawn to distinguish significance level of diseases categories based on the use reports of its sub categories treated with medicinal plant species. All analyses were run in CANOCO 5 (11, 12).

## Results

### Demography

Interviews were conducted from different fields of life viz; local traditional practitioners, veterinary doctors, farmers, and other local respondents. The current study showed that farmers have a preference to collect medicinal plants directly from the field, since they can easily collect and use the plants. Each village of the study area has many expert persons in livestock treatment. The survey showed that most of the people have some information about the use of herbal medicine for the treatment of their livestock, where this information is usually passed down from their parents or elders.

### Floristic Contribution in Ethnoveterinary Practices

A total of 50 medicinal plants belonging to 38 families have been reported by the local respondents (Table 1), where Poaceae was the most cited family. In these ethnoveterinary medicinal plant species, 50% were herbaceous growth habit, 28% were trees, and 22% were shrubs. Out of these 50% medicinal plants, 70% were wild in nature and 30% were cultivated (Table 2). All of the understory medicinal plant species were used for curing various veterinary ailments. The common livestock diseases are red water (Kalangari), 3 days sickness (Taqo), diarrhea (Reekh), tympany (parsob), and indigestion (Charmekh) among others (Table 1).

**Table 1:** Complete information of medicinal plants used in curing veterinary diseases.

**S. no**	**Botanical name**	**Vernacular name**	**Family**	**Habit**	**Part used**	**Carrier used for the dosage**	**Used alone or in combination with other plants**	**Disease for which it is used**	**Special care (Parhez)**
1	*Acacia modesta*	Polosa	Mimosaceae	T	G	Mixed with flour	Mixed with other plants	Three days sickness, indigestion	Cold water, bitter things
2	*Acorus calamus*	Bekan	Acoraceae	H	R	Mixed with flour	Alone	Abdominal	Nil
3	*Aesculus indica*	Jooz	Hippocastanaceae	T	F	Mixed with flour	Alone	Abdominal pain	Cold water
4	*Allium cepa*	Piaz	Alliaceae	H	B	Alone	Alone	Indigestion, tymphany	Nil
5	*Allium sativum*	Ooga	Alliaceae	H	B	Alone	*Capscum annuum*	Fever	Cold water
6	*Aloe vera*	Manzarepanra	Liliaceae	H	L	Mixed with flour	Alone	Weakness, paralysis	–
7	*Berberis lycium*	Ziar large (Kware)	Berberidaceae	S	B, W	Oil	Alone	Wound, weakness, fever	–
8	*Bombax ceiba*	Badar	Bombacaceae	T	B	Mixed with Milk	Alone	For fracture	Pregnant animals
9	*Brassica campestris*	Sharsham	Brassicaceae	H	S	Alone	Alone	For body temperature	Nil
10	*Brassica nigra*	Thor sharsham	Brassicaceae	H	P	Alone	Alone	Placenta retention	Nil
11	*Butea monosperma*	Shanaipalai	Papilionaceae	T	Fs	Mixed with flour	Alone	Constipation	Nil
12	*Buxus wallichiana*	Shamshad	Buxaceae	T	L	Used in water suspension	Alone	Liver fluke in liver	Nil
13	*Calotropis procera*	Spalmai	Asclepiadaceae	S	L	Mixed with flour	*Trachyspermumammi*	Three days sickness, indigestion	From cold things, from green grass
14	*Cannabis sativa*	Bhang	Moraceae	H	L	Alone	Alone	Red water	Nil
15	*Capsicum annuum*	Marchakay	Solanaceae	H	F	Alone	*Oxalis acetosella*	Fever	From hotness, from sun
16	*Cedrella serrate*	Meem	Meliaceae	T	L	Mixed with flour	*Acorus calamus*	Fever	Nil
17	*Cissampelos pareira*	Tangapanra	Menispermaceae	H	L	Used in water suspension	Alone	Cough	Nil
18	*Citrullus colocynthis*	ZangaliHindwana	Cucurbitaceae	H	F	Mixed with flour	Alone	Abdominal pain, defection	From cold water
19	*Daphne oleoides*	Kotilal	Thymelaeaceae	S	Rs	Mixed with flour	Alone	Weakness, disease in milk production	From cold things
20	*Diospyros lotus* L.	Tor amluk	Ebenaceae	T	F	Mixed with flour	Alone	Diarrhea	Nil
21	*Dodonaea viscosa*	Ghawarasky	Sapindaceae	S	L	Alone	Alone	Fracture	From walking
22	*Equisetum arvensis*	Bandakey	Equisetaceae	H	St	Alone	Alone	Red water	Nil
23	*Ficus carica*	Inzar	Moraceae	T	L	Mixed with flour or alone	Alone	Placenta retention	Nil
24	*Foeniculum vulgare*	Kaga	Apiaceae	H	L	Alone	Alone	Weakness, anorexia	Nil
25	*Grewia optiva*	Pastaone	Tiliaceae	T	B	Mixed with flour	Alone	Round worm, tap worms, liver fluke, placenta retention	Nil
26	*Hordeum vulgare*	Warbashe	Poaceae	H	S (Powdered)	Alone	Sand	If animal eat a local insect	Nil
27	*Indigofera heterantha*	Keenthai	Fabaceae	S	R	Alone	Alone	Abdominal pain	Nil
28	*Litsea cubeba*	Khadang	Lauraceae	T	B	Mixed with Halwa (desert)	Alone	Wound, fracture	Nil
29	*Mallotus philippensis*	Kambela	Euphorbiaceae	T	S	Mixed with flour	Alone	Liver fluke	Cold water, green grass
30	*Melia azedarach*	Bakyanra	Meliaceae	T	F	Mixed with flour	Alone	Disease in milk production	Nil
31	*Mentha longifolia*	Welani, inale	Lamiaceae	H	L	Mixed with flour	*Trachyspermumammi*	Dried muzzle, 3 days sickness	Nil
32	*Musa paradisiaca*	Kela	Musaceae	T	L	Alone	Alone	Red water	Nil
33	*Opuntia dillenii*	Zuqam	Cactaceae	H	F	Alone	Alone	Wound in eyes	Nil
34	*Origanum vulgare*	Shamke	Lamiaceae	H	St, L	Alone	Alone	Fever	Nil
35	*Oxalis acetosella*	Tarooke	Oxalidaceae	H	P	Alone	Alone	Red water, itching	From hot things
36	*Paeonia emodi*	Mamekh	Paeoniaceae	H	R	Mixed with flour	Alone	Wound, weakness	Nil
37	*Pinus roxburghii*	Nakhtar	Pinaceae	T	Bn	Mixed with flour	*Berberis lycium*	Red water	From cold water
38	*Podophyllum emodi*	Kakora	Podophyllaceae	H	R	Mixed with flour	Alone	Constipation, diarrhea	Nil
39	*Prunus persica*	Shaltallo	Rosaceae	H	Leave	Used in water suspension	Alone	Maggots wound	–
40	*Pyrus pashia*	Tango	Rosaceae	H	S	Alone	Alone	Wound in eyes	Nil
41	*Rumex dentatus*	Shalkhe	Amaranthaceae	H	R	Mixed with Milk	Alone	Wound, tymphany, diarrhea	Nil
42	*Solanum* surattense	Mara ghone	Solanaceae	H	L	Alone	Alone	Paralysis	Nil
43	*Trachyspermum ammi*	Speerkay	Apiaceae	H	S	Mixed with flour	Alone	Temphany, all pain, anorexia	Nil
44	*Trigonella foenumgracium*	Malkhawaze	Papilionaceae	H	S	Mixed with flour	Alone	Fever, decrease in milk production	From cold water
45	*Triticum aestivum*	Ghanam	Poaceae	H	S	Alone	*Berberis lycium*	Wound	–
46	*Tylophora hirsuta*	Goganda	Asclipidaceae	H	R	Mixed with flour	Alone	Weakness, diarrhea, tymphany	From cold water
47	*Vitis vinifera*	Angoor	Vitaceae	H	St	Mixed with flour	Mixed with other plant	Red water	Nil
48	*Verbascum thapsus*	Khardag	Verbenaceae	H	R	Mixed with flour	Alone	Diarrhea, abdominal pain	Nil
49	*Zanthoxylum armatum*	Dambara	Asteraceae	H	F	Mixed with flour	Alone	Three days sickness, fever	Nil
50	*Zea mays*	Jawar	Poaceae	H	S	Alone	Alone	Diarrhea	Nil

*Abbreviations for plant habit: H, Herb; S, Shrub; T, Tree; R*.

*Abbreviations for part used: Root; F, Fruit; S, Seed; St, Stem; Wp, Whole plant; L, Leaves; W, Wood; G, Gum; Bn, Branch with needle; W, Wild; C, Cultivated; S*.

*Abbreviations for cultivation method: Seed; St, Stem; R, Root; P, Plant; Bd, Buds*.

**Table 2:** Market availability, status, nature, part for cultivation, and therapeutic practices of the medicinal plants recorded from Chamla Valley.

**S. no**	**Botanical name**	**Is this plant sold in local market?**	**Conservation status**	**Nature of plant**	**Part used for cultivation**	**From how long the medicinal properties have been known (years)?**
1	*Acacia modesta*	Yes	Change	Wild	S	40
2	*Acoruscalamus*	Yes	No change	Wild	R	50
3	*Aesculus indica*	No	No change	Wild	S	50
4	*Allium cepa*	Yes	Increase	Cultivated	P	45
5	*Allium sativum*	Yes	Increase	Cultivated	P	35
6	*Aloe vera*	No	No change	Cultivated	Bd	40
7	*Berberis lycium*	Yes	Decrease	Wild	R	44
8	*Bombaxceiba*	No	Increase	Cultivated	P	50
9	*Brassica campestris*	Yes	Increase	Cultivated	S	45
10	*Brassica nigra*	No	No change	Wild	S	50
11	*Butea monosperma*	Yes	No change	Wild	S	43
12	*Buxus wallichiana*	No	Decrease	Wild	S	50
13	*Calotropis procera*	No	No change	Wild	S	40
14	*Cannabis sativa*	No	No change	Wild	S	50
15	*Capsicum annuum*	Yes	Increase	Cultivated	S	50
16	*Cedrella serrata*	No	Decrease	Wild	S	55
17	*Cissampelos pareira*	No	Decrease	Wild	R	49
18	*Citrullus colocynthis*	No	Decrease	Wild	S	50
19	*Daphne oleoides*	Yes	Decrease	Cultivated	S	55
20	Tylophora hirsuta	No	Decrease	Wild	S	44
21	*Dodonaea viscosa*	No	Decrease	Wild	S	50
22	*Equisetum arvensis*	No	No change	Wild	R	40
23	*Ficus carica*	No	Decrease	Wild	S	50
24	*Foeniculum vulgare*	No	No change	Wild	S	55
25	*Grewia optiva*	Yes	Increase	Wild	S	49
26	*Hordeum vulgare*	No	Decrease	Cultivated	S	45
27	*Indigofera heterantha*	Yes	Decrease	Wild	S	40
28	*Litsea cubeba*	Yes	Decrease	Wild	R	15
29	*Mallotus philippensis*	No	Decrease	Wild	S	44
30	*Melia azedarach*	No	Increase	Wild	S	55
31	*Mentha longifolia*	No	Decrease	Wild	R	25
32	*Musa paradisiaca*	No	Increase	Cultivated	R	45
33	*Opuntia dillenii*	No	Increase	Wild	St	40
34	*Origanum vulgare*	No	No change	Wild	R	50
35	*Oxalis acetosella*	No	No change	Wild	S, R	45
36	*Paeonia emodi*	Yes	Decrease	Wild	R	55
37	*Pinus roxburghii*	No	Decrease	Wild	S	45
38	*Podophyllum emodi*	No	Decrease	Wild	S	50
39	*Prunus persica*	No	Increase	Wild	S	40
40	*Pyrus pashia*	No	No change	Wild	S	39
41	*Rumex dentatus*	No	Decrease	Wild	S, R	45
42	*Solanum* surattense	No	No change	Wild	S	40
43	*Trachyspermumammi*	Yes	Increase	Cultivated	S	40
44	*Trigonella foenumgracium*	Yes	Decrease	Cultivated	S	50
45	*Triticum aestivum*	Yes	Increase	Cultivated	S	45
46	*Tylophora hirsuta*	No	Decrease	Wild	S	46
48	*Vebascum thapsus*	No	No change	Wild	St	50
47	*Vitis vinifera*	No	Increase		S	30
49	*Zanthoxylum armatum*	Yes	Decrease	Wild	S	35
50	*Zea mays*	Yes	Increase	Cultivated	S	50

*Abbreviations part used for cultivation: S, Seed; St, Stem; R, Root; P, Plant; Bd, Buds*.

Most of the plants are used in fresh condition. According to the results, in some cases for a single disease many plants were used. Some medicinal plants are used for single disease, such as *Canabis sativa, Cedrella serrate*, and *Origanum vulgare* were used for fever, *Brassica nigra* was used for placenta retention, *Butea monosperma* for constipation. On the other hand, species cited for multi disorders were *Berberis lycium* for wound, weakness, and fever, *Acacia modesta, Calotropis procera* for 3 days sickness and indigestion, *Daphne* oleoides for weakness and disease in milk production, *Grewia optiva* for round worm, tap worms, liver fluke, and placenta retention, *Trigonella foenumgracium* for fever, decrease in milk production (Table 1). In addition, we provide some chemical compounds present in some plant species used by local respondents (see Table 3).

**Table 3:** Chemical compounds present in some plant species used by local respondents of Chamla Valley, KP, Pakistan.

**Species name**	**Chemical compound**	**References**
*Aesculus indica* (Wall. ex Camb.) Hook	Aesin, decanoic acid, quercitin, saponins	(13, 14)
*Berberis lycium* Royle	Berberine, berbamine, punjabine	(15, 16)
*Cannabis sativa* L.	Cannabigerol, cannabidiol	(17, 18)
*Grewia optiva* J.R.Drumm. ex Burret	Grewialin, optivanin	(19, 20)
*Indigofera heterantha* Brandis	Lactone, flavonides, glycosides, saponins	(21)
*Ficus carica* L.	Cyaniding, furanoid, cinnamic alcohol, eugeno, flavonols, ficusin	(22–25)
*Pyrus pashia* Buch.-Ham. ex D.Don	Flavanoids, saponins. coumarins, terpenes	(26, 27)
*Origanum vulgare* L.	Thymol, linalool, salvianolic, lithospermic, syringic, caffeic acids	(28–30)
*Verbascum thapsus* L.	Aucubin, flavonoids, saikogenin,saponins	(31, 32)
*Zanthoxylum armatum* DC.	Linalool, palmitoleic acid	(33, 34)

### Carrier Used for the Dosage

The local community members used different carriers for making the traditional medicine, i.e., flour, water, oil, milk, halwa (a traditional dessert) to treat the diseases. Out of 50 medicinal plants, 24 are used with flour (Table 1). It is important to mention that these ethnoveterinary approaches are practiced from more than five decades (25–55 years) (Table 4).

**Table 4:** Quantitative analysis of the medicinal plants of Chamla Valley, KP, Pakistan.

**S. no**	**Species name**	**Basic values**			**Indices**	
		**U_**i**_**	**N**	**FC**	**UVi**	**RFCs**
1.	*Acacia modesta*	25	39	25	0.64	0.21
2.	*Acorus calamus*	30	42	30	0.71	0.25
3.	*Aesculus indica*	17	32	17	0.53	0.14
4.	*Allium cepa*	15	36	15	0.42	0.13
5.	*Allium sativum*	32	41	32	0.78	0.27
6.	*Aloe vera*	12	33	12	0.36	0.10
7.	*Berberis lycium*	8	37	8	0.22	0.07
8.	*Bombaxceiba*	25	39	25	0.64	0.21
9.	*Brassica campestris*	28	35	28	0.80	0.23
10.	*Brassica nigra*	18	41	18	0.44	0.15
11.	*Buteamonosperma*	40	48	40	0.83	0.33
12.	*Buxus wallichiana*	16	40	16	0.40	0.13
13.	*Calotropis procera*	31	42	31	0.74	0.26
14.	*Cannabis sativa*	16	38	16	0.42	0.13
15.	*Capsicum annuum*	29	35	29	0.83	0.24
16.	*Cedrella serrata*	11	41	11	0.27	0.09
17.	*Cissampelos pareira*	13	38	13	0.34	0.11
18.	*Citrullus colocynthis*	18	36	18	0.50	0.15
19.	*Daphne oleoides*	33	42	33	0.79	0.28
20.	*Diospyros lotus* L.	13	30	13	0.43	0.11
21.	*Dodonaea viscosa*	29	37	29	0.78	0.24
22.	*Equisetum arvensis*	26	43	26	0.60	0.22
23.	*Ficus carica*	24	38	24	0.63	0.20
24.	*Foeniculum vulgare*	32	45	32	0.71	0.27
25.	*Grewia optiva*	16	36	16	0.44	0.13
26.	*Hordeum vulgare*	19	40	19	0.48	0.16
27.	*Indigofera heterantha*	12	36	12	0.33	0.10
28.	*Litsea cubeba*	7	39	7	0.18	0.06
29.	*Mallotus philippensis*	35	42	35	0.83	0.29
30.	*Melia azedarach*	23	32	23	0.72	0.19
31.	*Mentha longifolia*	29	41	29	0.71	0.24
32.	*Musa paradisiaca*	22	31	22	0.71	0.18
33.	*Opuntia dillenii*	13	30	13	0.43	0.11
34.	*Origanum vulgare*	19	38	19	0.50	0.16
35.	*Oxalis acetosella*	14	35	14	0.40	0.12
36.	*Paeonia emodi*	20	34	20	0.59	0.17
37.	*Pinus roxburghii*	18	40	18	0.45	0.15
38.	*Podophyllum emodi*	21	39	21	0.54	0.18
39.	*Prunus persica*	29	42	29	0.69	0.24
40.	*Pyrus pashia*	17	37	17	0.46	0.14
41.	*Rumex dentatus*	15	33	15	0.45	0.13
42.	*Solanum* surattense	13	38	13	0.34	0.11
43.	*Trachyspermum ammi*	11	35	11	0.31	0.09
44.	*Trigonella foenumgracium*	20	44	20	0.45	0.17
45.	*Triticum aestivum*	24	41	24	0.59	0.20
46.	*Tylophora hirsuta*	16	33	16	0.48	0.13
47.	*Vitis vinifera*	24	35	24	0.69	0.20
48.	*Verbascum thapsus*	12	23	12	0.52	0.10
49.	*Zanthoxylum armatum*	7	29	7	0.24	0.06
50.	*Zea mays*	24	45	24	0.53	0.20

### Special Care (Parhez)

Most of the plants are used in fresh condition and those plants that are used in dry condition are at the top of the mountains and they cannot be obtained immediately. In addition, most of these plants are not available in the market. Plants are mostly given singly and usually there is no special care in the treatment process, but few plants need special care, locally called as “parhez” (Table 1). Generally, effects of the drugs are mostly quick and the common animal diseases are red water, 3 days sickness, diarrhea, tympani, and indigestion.

### Use Value (UVi) and Relative Frequency Citations (RFCs)

The UVi ranged from 0.18 to 0.83 (Table 4). The plants with highest value of UVi were *Butea monosperma* and *Mallotus philippensis* (UVi = 0.83), followed by *Brassica campestris* (0.80), *Daphne* oleoides (0.79), *Allium sativum*, and *Dodonaea viscosa* (both with 0.78), respectively. On the other hand, the plant with the lowest value was *Litsea cubeba* (0.18). Relative frequency citation ranged from 0.06 to 0.33 (Table 4). Based on the RFC values, the most valuable and cited medicinal plant species by the traditional practitioners and local respondents were *Butea monosperma* (RFCs = 0.33), *Mallotus philippensis* (0.29) and *Daphne oleoides* (0.28). The plants with the lowest RFCs were *Litsea cubeba* and *Zanthoxylum armatum* (RFCs = 0.06).

### Principle Components Analysis (PCA)

**Figure 1:**
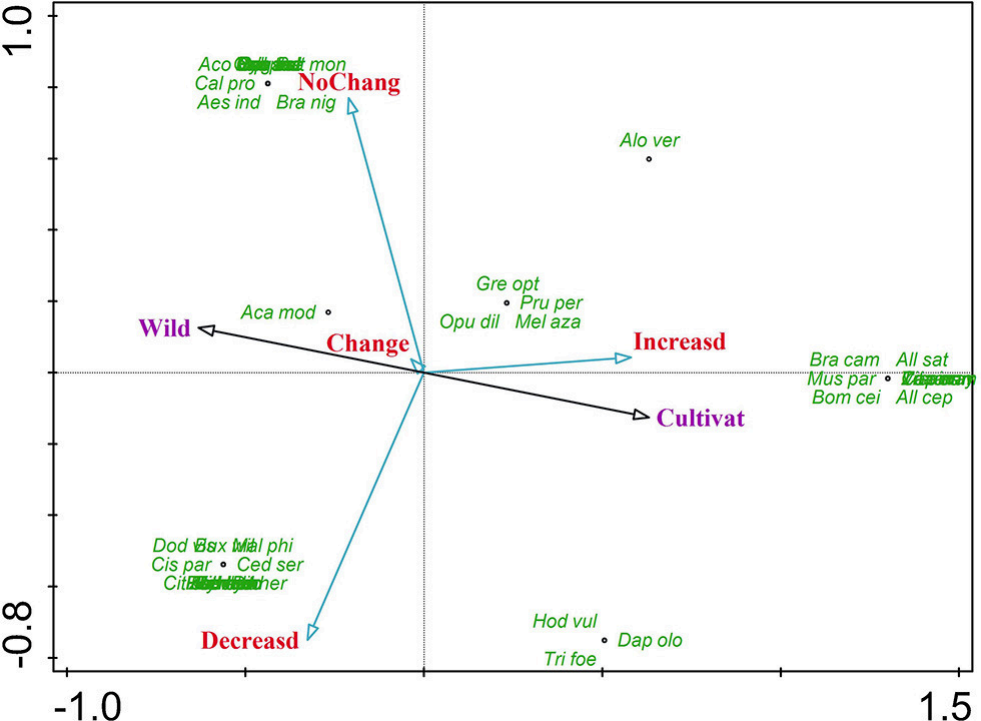
Principle component analysis showing the correlation between plant species and their variables.

Principle components analysis (PCA) was determined to examine the correlation between plant species and treated ailments (disease categories). The PCA results revealed that *Allium cepa, Allium sativum, Bombax ceiba, Brassica campestris, Capsicum annuum*, and *Melia azedarach* were most frequently found with positive and significant correlation with increased population; and *Buxus wallichiana, Cedrella serrata, Cissampelos pareira, Citrullus colocynthis, Daphne oleoides*, and Tylophora hirsuta was cited as decreased species. *Acacia modesta, Acorus calamus, Aesculus indica*, and *Berberis lycium* were positive and significant in correlation with wild nature and all these species were most frequently found in wild habitats. *Allium cepa, Allium sativum*, and *Aloe vera* were found cultivated. All the remaining species-diseases correlations are illustrated in Figure 1.

### Species Response Curve (SRC)

**Figure 2:**
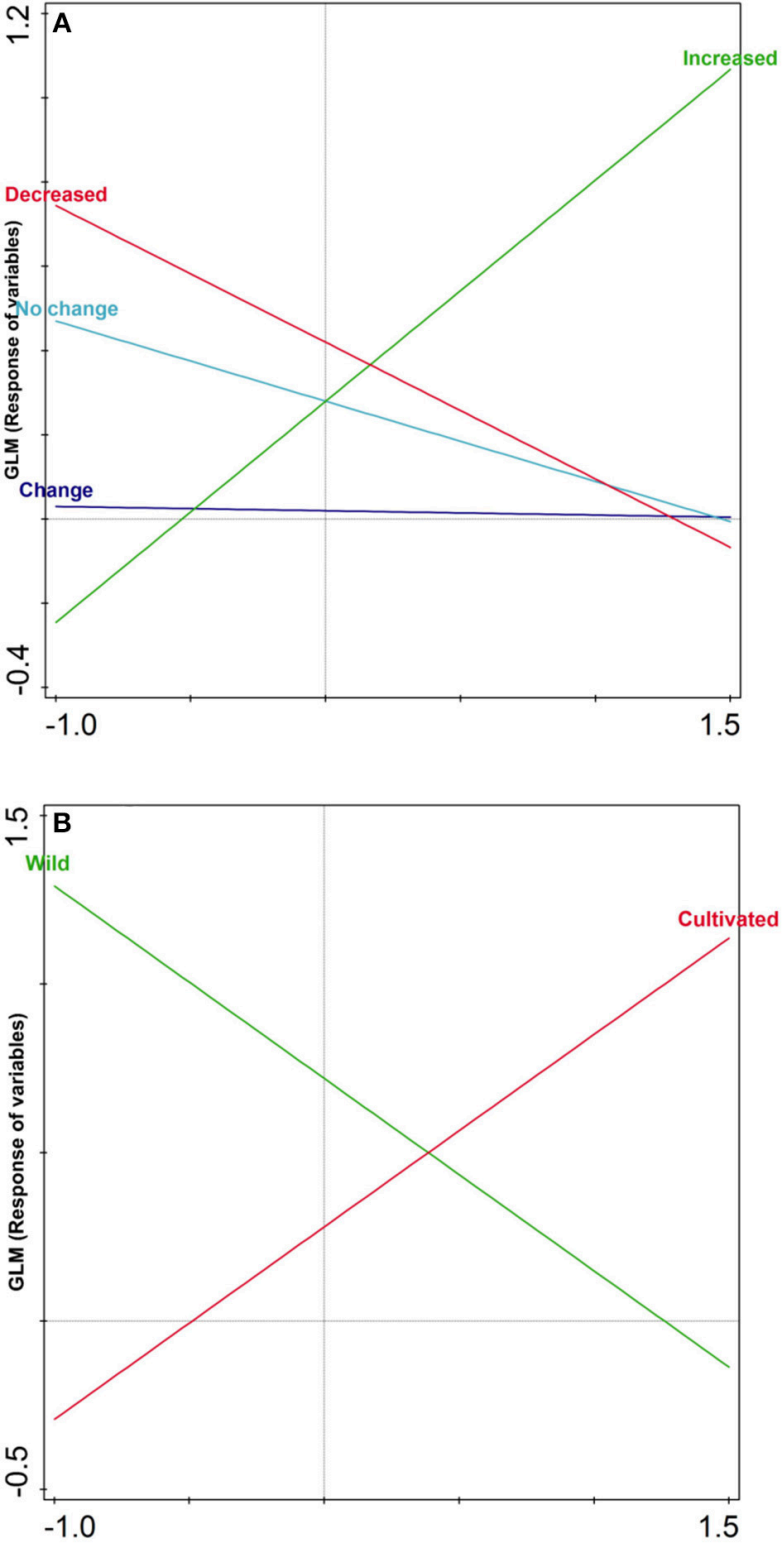
Species response curve shows the significance level among various variables conservation status **(A)** and nature of plant **(B)** on the basis of their frequency.

The analysis clearly indicates highly significant differences (*p* < 0.00001) for increased population variable (conservation status) in comparison with all other variables due to their maximum frequency as shown in Figure 2A. Additionally, this disease category also showed maximum response (72.5%) and computed value (*F* = 126.8) as well. Nevertheless, wild and cultivated frequency of species (Figure 2B) also showed highly significance (*p* < 0.00001) due to its citations frequency with a response percentage (85.8%) and (85.8%) and computed value (*F* = 289.9) and value (*F* = 289.9), respectively. Furthermore, the remaining variables showed non-significant differences as mentioned in the Table 5.

**Table 5:** Summary of fitted Generalized Linear Models 6 response variables.

**Response**	**Type**	***R*^**2**^[%]**	***F***	***p***
Change	linear	0.3	0.1334	0.71649
No change	linear	9.5	5.1	0.02907
Increased	linear	72.5	126.8	< 0.00001
Decreased	linear	22.9	14.3	0.00043
Wild	linear	85.8	289.9	< 0.00001
Cultivated	linear	85.8	289.9	< 0.00001

### Status of Ethnoveterinary Medicinal Plants

At high altitude, high diversity in medicinal plants population was found. While, as we go toward the plain area, the number of species decreases. The population of most of medicinal plants has been decreased in last 30 years (Table 4). Only few plants, which are cultivated or not grazed by animals, are increased.

## Discussion

### Floristic Medicinal Contribution

Pakistan has a wide variety of flora and fauna, where flora contains about 6,000 species of phanerogams (1). In Pakistan the local populations of different regions have century's old knowledge and traditional practices of most of the plants occurring in those regions. This local knowledge of plants has been transferred from generation to generation through verbal communication and personal experience. The local people utilized locally available herbal recipes due to many reasons e.g., the people are poor and cannot afford modern veterinary medicine and there is a long distance between their residence and the area where some modern veterinary facilities are available. Due to this reason the traditional herbal medicine is the first option for the people of Chamla Valley (Study area). Harun-or-Rashid et al. (35) reported from Bangladesh that lack of access to modern veterinary facilities and because of the high prices of medicines, most farmers rely on traditional healers for cure of livestock diseases. Due to this reason the traditional herbal medicine is the first option of the people of Chamla Valley and they depend on medicinal plants to cure various diseases of human and animals.

In the present study, herbal healers and local respondents reported a total of 50 medicinal plants used in the treatment of livestock diseases. These medicinal plants are used for various ailments. Some medicinal plants are used for single disease, while some are used for many diseases. Also species cited for multi disorders were *Acacia modesta, Calotropis procera* for 3 days sickness and indigestion, *Daphne oleoides* for weakness and disease in milk production, *Grewia optiva* for roundworm, tap worms, liver fluke, and placenta retention. Similarly, Jabbar et al. (36) and Tabassam et al. (37) have reported different plant species used in animal treatment from different parts of Pakistan. Dilshad et al. (38) has reported 66 plant species from Sargodha district of Pakistan and Farooq et al. (39) reported 18 plant species representing 14 families to cure parasitic diseases of livestock from Cholistan desert of Pakistan. In another similar study in Mansehra district, a selected hilly area of Pakistan, Sindhu et al. (40) reported 35 plant species belonging to 25 families [also see Ole-Midron (41)].

### Use Value and Relative Frequency Citations

The medicinally used plants with highest use values were *Butea monosperma, Mallotus philippensis, Brassica campestris* and *Daphne oleoides*. Greater use values of these mentioned medicinal plants might be due to their widespread distribution and also due to local practitioners' awareness, which makes those plants as the first choice for ailment (11). Based on the RFC values, the most valuable and cited medicinal plant species by the traditional practitioners and local respondents were *Butea monosperma, Mallotus philippensis*, and *Daphne oleoides*. Maximum relative frequency citations clarify the facts that the cited plants species are well familiar to the number of traditional drivers (42) and they should be further evaluated in pharmacognostic studies (43).

### Conservation of Medicinal Plants

The number of ethnoveterinary medicinal plant species is decreasing, as showed by this study. One reason for this is the excessive and unwise utilization, over grazing, climate change, increase in population, poor method of collection like dig out the whole plant, market pressure and deforestation. The local people cut down the forests for the cultivation of orange fruits and other plants because it is the high source of income for local people. Some plants are collected from the mountains, brought to local market by the local people and then transferred to the major cities. The wild plants are seriously endangered. Some plants are only present only at high altitude such as Maban area while in lower mountains they had been vanished and extinct. Local people want to increase their area of cultivation and for this purpose they burn the whole forest, affecting the whole plant population. For instance, besides being cut off indiscriminately, the seedlings of the *Pinus* were also destroyed during burning of the forest.

The existence of forest is essential for the life of these medicinal plant species, an awareness program in the region about the status of indigenous flora, sustainable plants collection and conservation of important medicinal plants will yield better outcomes (9). The indigenous community should be involved in conservation practices and the local staff, local stakeholders, and plant collectors should be aware about the conservation of plant resources of the area. Sher et al. (44) indicated that the investigated area of district Buner is under heavy deforestation, biotic interference, and overgrazing pressure. Resultantly, valuable economic and medicinal plants of the area are reducing. Sustainable utilization, suitable management and conservation of the flora of the area are highly suggested.

## Conclusions

This is the first study to collect and organize data about the medicinal plants that are used in the treatment of livestock diseases and identify those species of plants that are endangered due to indiscriminate usage in Buner district, Pakistan. The results showed that (i) the most common livestock diseases are red water, tymphany, constipation, diarrhea, fever, and indigestion, (ii) *Butea monosperma, Mallotus philippensis, Brassica campestris*, and *Daphne oleoides* are the most representative medicinally plant species, and (iii)*Paeonia emodi, Berberis lyceum*, and *Pinus roxburghii* are facing very strong pressure due to their indiscriminate harvesting by the local people.

Therefore, we suggest that there is dire need to protect forest and conserve the habitats for flora and fauna. For this, government and NGOs need to implement strong programs with the participation of local people, which need to be made aware of the importance of conserving the precious forest resources and taking part in the plantation for future generations.

## Author Contributions

KK and NA conceived designed the experiment. KK performed the experiment. IUR analyzed the data. KK, IUR, and NA wrote the manuscript. EC and FI commented and made the final suggestions in the manuscript.

### Conflict of Interest Statement

The authors declare that the research was conducted in the absence of any commercial or financial relationships that could be construed as a potential conflict of interest.
